# Increased Rate of Retinal Pigment Epithelial Cell Migration and Pro-Angiogenic Potential Ensuing From Reduced Cystatin C Expression

**DOI:** 10.1167/iovs.61.2.9

**Published:** 2020-02-12

**Authors:** Emil Carlsson, Wasu Supharattanasitthi, Malcolm Jackson, Luminita Paraoan

**Affiliations:** 1 Department of Eye and Vision Science, Institute of Ageing and Chronic Disease, University of Liverpool, Liverpool, United Kingdom; 2 Department of Physiology, Faculty of Pharmacy, Mahidol University, Bangkok, Thailand; 3 Department of Musculoskeletal Biology, Institute of Ageing and Chronic Disease, University of Liverpool, Liverpool, United Kingdom

**Keywords:** Cell migration, retinal pigment epithelium, cystatin C, age-related macular degeneration

## Abstract

**Purpose:**

Variant B precursor cysteine protease inhibitor cystatin C, a known recessive risk factor for developing exudative age-related macular degeneration (AMD), presents altered intracellular trafficking and reduced secretion from retinal pigment epithelial (RPE) cells. Because cystatin C inhibits multiple extracellular matrix (ECM)–degrading cathepsins, this study evaluated the role of this mutation in inducing ECM-related functional changes in RPE cellular behavior.

**Methods:**

Induced pluripotent stem cells gene-edited bi-allelically by CRISPR/Cas9 to express the AMD-linked cystatin C variant were differentiated to RPE cells and assayed for their ability to degrade fluorescently labeled ECM proteins. Cellular migration and adhesion on multiple ECM proteins, differences in transepithelial resistance and polarized protein secretion were tested. Vessel formation induced by gene edited cells–conditioned media was quantified using primary human dermal microvascular epithelial cells.

**Results:**

Variant B cystatin C–expressing induced pluripotent stem cells–derived RPE cells displayed a significantly higher rate of laminin and fibronectin degradation 3 days after seeding on fluorescently labeled ECM (*P* < 0.05). Migration on matrigel, collagen IV and fibronectin was significantly faster for edited cells compared with wild-type (WT) cells. Both edited and WT cells displayed polarized secretion of cystatin C, but transepithelial resistance was lower in gene-edited cells after 6 weeks culture, with significantly lower expression of tight junction protein claudin-3. Media conditioned by gene-edited cells stimulated formation of significantly longer microvascular tubes (*P* < 0.05) compared with WT-conditioned media.

**Conclusions:**

Reduced levels of cystatin C lead to changes in the RPE ability to degrade, adhere, and migrate supporting increased invasiveness and angiogenesis relevant for AMD pathology.

The retinal pigment epithelium (RPE) comprises a unique and critical tissue in the posterior of the eye responsible for multiple aspects of maintaining visual function.^1^ Of clinical relevance, dysfunctional and progressive loss of RPE is one of the hallmarks of late-stage age-related macular degeneration (AMD),^2^^,^^3^ leading to loss of central vision. This is the leading form of blindness in the elderly, but the underlying molecular mechanisms are poorly understood.

RPE cells form a tightly packed, highly polarized monolayer between the photoreceptors and Bruch’s membrane (BrM), which in turn separates it from the choroidal blood supply. The RPE exhibits differential secretion of key effector proteins that are essential for retinal health.^4^ One of these is the cystatin C, a 120 amino acid long protease inhibitor known to act on cysteine proteases such as cathepsins B, H, and L.^5^ The cysteine cathepsin family is comprised of 11 proteins—cathepsin B, C, F, H, K, L, O, S, V, W, and X,^6^ which are secreted in high levels to the extracellular space where they are major players in extracellular matrix (ECM) remodeling.^7^^,^^8^ Chronic elevation of cathepsin activity is associated with inflammatory disease^9^ and can be caused by an imbalance with their endogenous inhibitors.^10^^,^^11^

Multiple studies have shown that cystatin C is among the most abundantly expressed proteins in the RPE,^12^^–^^14^ highlighting the importance of its proteolytic regulatory function in the retina. A single G73A point mutation in the cystatin C gene (CST3) has been shown to result in an increased risk for development of AMD^15^^,^^16^ and Alzheimer's disease,^15^^,^^17^^–^^20^ two neurodegenerative diseases that share multiple characteristics on a molecular level.^21^ The mutation results in a variant of the protein designated variant B, which encodes an A25T amino acid substitution in the penultimate position of the highly hydrophobic secretory signal leader sequence of the precursor cystatin C. Because this leader region of pre-cystatin C is cleaved off before the mature form of the protein is secreted, this mutation does not affect the function of the mature protein. However, the variant B recessive genotype has been shown to reduce the rate of cystatin C secretion,^22^^,^^23^ possibly as a result of intracellular mis-trafficking where an intracellular pool of misprocessed protein associates with mitochondria.^23^

A bottleneck in the study of RPE function has traditionally been the lack of source material, leading investigators to use spontaneously immortalized cell lines, such as ARPE19, which in some ways mimic the function of primary RPE cells.^24^ Recent advances in the field of stem cell differentiation have led to the establishment of protocols for both spontaneous and targeted differentiation toward RPE fate.^25^ Combined with gene-editing techniques such as CRISPR/Cas9, this provides an excellent platform for studying the effect of specific protein variants in RPE cells, defining a strategy that was also used in this study.

Our study shows that iPS-derived RPE cells gene-edited on both alleles (to reproduce the recessive disease-associated phenotype) by CRISPR/Cas9 to express the less-abundant variant B form of cystatin C exhibit abnormal cellular migration and adhesion to common ECM proteins compared with wild-type (WT) cystatin C–expressing cells. The gene-edited cells also degraded fluorescently labeled laminin and fibronectin deposited into NIH3T3 fibroblast ECM 3D matrices significantly more quickly than nonedited cells, and although the gene-edited iPS-RPE cells formed monolayers with transepithelial resistance (TER) notably higher than the commonly used RPE cell line ARPE19, these values were significantly lower than those achieved by the nonedited cells.

## Methods

### Cell Culture

Human-induced pluripotent stem cells (iPSCs) were obtained from the Human Induced Pluripotent Stem Cells Initiative and were cultured in a feeder-free system on six-well plates coated with recombinant human truncated vitronectin (Thermo Fisher Scientific, Waltham, MA) in Essential-8 medium (E8; Thermo Fisher Scientific) at 37° C, 5% CO_2_. Cells were routinely passaged by dissociation with Versene (Thermo Fisher Scientific) at a 1:6 split ratio. Human ARPE19 cell line (P25) was cultured in T75 flasks in Dulbecco's modified Eagle’s medium/nutrient mixture F-12 Ham (DMEM; F12; Sigma-Aldrich, St. Louis, MO) supplemented with 10% fetal bovine serum (FBS; Sigma-Aldrich) at 37°C, 5% CO_2_. Mouse NIH3T3 fibroblast cell line (ECACC) was cultured in T75 flasks in high glucose DMEM (Sigma-Aldrich) supplemented with 10% FBS at 37° C, 5% CO_2_. ARPE19 and NIH3T3 cells were routinely passaged with trypsin-EDTA solution (Sigma-Aldrich) and split at a 1:3–1:10 ratio. Human dermal microvascular epithelial cell (PromoCell, Heidelberg, Germany) and normal human dermal fibroblasts (NHDFs; PromoCell) were cultured in gelatin-coated T75 culture flasks in Endothelial Cell Growth Medium V2 (PromoCell) or Fibroblast Growth Medium (PromoCell), respectively, at 37° C, 5% CO_2_. HDMECs and NHDFs were passaged using the PromoCell DetachKit and split at a 1:3–1:5 ratio.

### CRISPR/Cas9 Gene Editing

The iPSCs homozygous for variant B cystatin C genotype were generated as previously described.^26^ CRISPR/Cas9 and donor sequences (including the G73A mutation in the CST3 gene) were synthesized by System Biosciences by subcloning the AGGGATAAAACCGCAGTCGC gRNA sequence, corresponding to a section of the CST3 gene upstream of the protein coding sequence, into the CAS701R-1 EF1-T7-hspCas9-T2A-RFP-H1-gRNA plasmid, and CST3 5′- and 3′-homology arms comprising approximately 800 bp were subcloned into the HR700PA plasmid. At 30 minutes before electroporation, WT iPSCs were treated with 10 µmol/L Rock inhibitor (Y-27632; Stemgent, Cambridge, MA). Cells were dissociated into single-cell suspension using Stempro Accutase (Thermo Fisher Scientific) and cotransfected with the CRISPR/Cas9 and donor constructs using the Neon electroporation system (Invitrogen, Carlsbad, CA) with parameters 1350V, 20 ms, 1 pulse. Cells were then seeded at clonal density and cultured in E8 medium for 3 days (supplemented with 10 µmol/L Rock inhibitor for the first day), followed by culture in Corning Nutristem hPSC XF medium (Thermo Fisher Scientific) under relevant antibiotic selective pressure for 1 week. Surviving clones were transferred manually to separate wells on 24-well plates using a P200 pipette and expanded in E8 medium. Genomic DNA was isolated using a DNeasy Blood & Tissue Kit (Qiagen, Hilden, Germany), and successful edits on both alleles were identified via PCR and confirmed by DNA sequencing performed by DNA Sequencing & Services (MRC I PPU; School of Life Sciences, University of Dundee, UK) using Applied Biosystems Big-Dye Ver 3.1 chemistry on an Applied Biosystems model 3730 automated capillary DNA sequencer. The selection cassette was then excised from the genome by LoxP-CRE recombination by transfecting the cells with CRE100A-1 plasmid (System Biosciences, Mountain View, CA), seeding at clonal density and isolating single clones as described above, followed by verification through DNA sequencing. Two separate gene-edited cell lines were generated from the same WT iPSC line and used in parallel throughout the study.

### Induced Pluripotent Stem Cell Differentiation to RPE

WT or gene-edited iPSCs were passaged at least once after thawing from cryostorage and seeded on matrigel-coated (BD Biosciences, Franklin Lakes, NJ) 6-well plates and grown until near confluence. Cells were differentiated toward RPE fate through a directed differentiation protocol using sequential exposure to nicotinamide and Activin A as previously described.^27^ Pigmented patches were excised with a scalpel and seeded at a minimum of 50,000 cells per cm^2^. For subsequent passaging, cells were dissociated with Stempro Accutase (Thermo Fisher Scientific).

### Measurement of Transepithelial Resistance

100,000 iPS-RPE cells were seeded per cm^2^ on transwell inserts on 12-well plates and cultured for up to 8 weeks. TER was measured weekly with a Millicell ERS-2 voltohmmeter (Merck, Kenilworth, NJ) by averaging three measurements per well and subtracting the value from a control well (containing media only) used as a baseline control. Final TER was calculated by multiplying the value with the surface area of the well (1.12 cm^2^).

### SDS-PAGE and Western Blot

Whole-cell lysates were prepared by homogenizing cells in lysis buffer (40 mmol/L Tris, pH 6.8, 10% glycerol, 1% SDS, 5% β-mercaptoethanol, 0.01% bromophenol blue) followed by passing through a G25 needle 10 times and boiling the sample at 95° C for 5 minutes. Conditioned media from transwell experiments was collected from the upper and lower chambers after 24 hours’ culture and normalized to equal volume with unconditioned culture media.

Proteins were resolved by electrophoresis on 12% acrylamide gels and transferred to nitrocellulose membranes (Sigma-Aldrich), which were blocked with 5% milk in TBS supplemented with 0.1% Tween-20 (Thermo Fisher Scientific) and probed with protein-specific antibodies. Antibodies used were against cystatin C (ab109508; Abcam, Cambridge, UK), MMP2 (4022; Cell Signaling Technology, Danvers, MA), pigment epithelium-derived factor (MAB1059; Millipore, Burlington, MA), RDH5 (ab101457; Abcam), α-tubulin (ab4074; Abcam), ZO1 (8193; Cell Signaling Technology), ZO2 (2847; Cell Signaling Technology), claudin-3 (214487; Abcam), integrin α4 (8440; Cell Signaling Technology) and integrin β1 (9699; Cell Signaling Technology). Proteins were finally detected by electrochemical luminescence using SuperSignal West Pico PLUS Chemiluminescent Substrate (Thermo Fisher Scientific) or Radiance Plus chemiluminescent substrate (Azure Biosystems, Dublin, CA) and imaged with a ChemiDoc XRS+ imaging system (Bio-Rad, Hercules, CA).

### Pluripotency Testing by Immunocytochemistry

The iPSC staining of selected pluripotency markers was performed as described previously.^26^ Briefly, iPSCs were fixed with 4% formaldehyde in PBS and probed for expression of SSEA4, OCT4, SOX2, and TRA-1-60 using a PSC 4-marker immunocytochemistry kit (Life Technologies, Carlsbad, CA), following manufacturer's instructions. Immunostained cells were imaged using a Zeiss Apotome (Zeiss, White Plains, NY) inverted fluorescence microscope.

### Cell Migration Assay

The iPS-RPE cells were cultured on 24-well plates coated with either matrigel, fibronectin (5 µg/cm^2^; Santa Cruz Biotechnology, Dallas, TX), laminin (5 µg/cm^2^; Santa Cruz Biotechnologies), or collagen IV (5 µg/cm^2^; Santa Cruz Biotechnologies) for 8 weeks. A wound approximately 500 µm wide was created by scratching the cell layer with a sterile 1000 µL pipette tip. Cells were then washed twice with DPBS (Dulbecco's phosphate-buffered saline) to remove detached cells and cultured for up to 1 month, with images of the wound recorded regularly using a Nikon Diaphot inverted light microscope (Nikon, Tokyo, Japan). The average size of the wound was analyzed by recording five separate fields per well and calculating the wound area in ImageJ,^28^ either using the MRI Wound Healing Tool (available at http://dev.mri.cnrs.fr/projects/imagej-macros/wiki/Wound_Healing_Tool) or by manually tracing the wound.

### Fluorescent Labeling of ECM Components

Recombinant human laminin or fibronectin 100 µg was labeled with a green fluorescent tag using an Alexa Fluor 488 microscale protein labelling kit (Thermo Fisher Scientific), following manufacturer's instructions. Fluorescently labelled proteins were stored in the dark at −20° C as 10-µg aliquots and used within 1 month of preparation.

### Analysis of Degradation of ECM Components

Predeposited ECM matrices were prepared using previously described methods.^29^^,^^30^ Briefly, NIH3T3 (ECACC 93061524) mouse fibroblast cells were cultured on matrigel-coated wells on 96-well plates in the presence of 5 µg/mL fluorescently labeled laminin or fibronectin. After 1 week, cells were washed with PBS, and plates were decellularized by treatment with 20 mmol/L NH_4_OH (Sigma-Aldrich) for 5 minutes, leaving the ECM still attached to the plate, and washed twice with PBS. Plates were sealed with parafilm and stored in the dark at 4° C for up to 1 week before being used for downstream application.

Matrix degradation by WT or gene-edited iPS-RPE cells was analyzed by seeding 100,000 cells per cm^2^ on denuded fluorescent matrices and culturing for up to 72 hours with images recorded of five separate fields per well every 24 hours using a Zeiss Apotome inverted fluorescence microscope. Total integrated fluorescence was calculated using ImageJ software. Matrix degradation was also determined after incubation with 3-day iPS-RPE conditioned media using the same method.

### Cell Adhesion Assay

The iPS-derived RPE cells were dissociated into single cell suspension using Stempro Accutase and suspended into serum-free DMEM at a concentration of 5 × 10^6^ cells/mL. Calcein AM (Cayman Chemical, Ann Arbor, MI) was added to achieve a final concentration of 5 µmol/L, and the cells were incubated at 37° C for 30 minutes. After being washed twice in DMEM, 100,000 cells were seeded per well on a black 96-well plate precoated with matrigel or 5 µg/cm^2^ laminin, collagen IV, or fibronectin. Plates were incubated at 37° C for 1 hour, followed by washing four times with DMEM, with separate wells representing 100% adhesion remaining unwashed. Fluorescence was then measured using a Glomax-Multi+ microplate reader (Promega, Madison, WI) equipped with a fluorescence module and blue optical kit (Ex/Em 490/510-570 nm).

### HDMEC/NHDF Co-Culture Angiogenesis Assay

In vitro co-culture assay of angiogenesis was adapted from a previously described protocol.^31^ Briefly, NHDFs (P5-10) were seeded on gelatin-coated wells on a 24-well plate at 20,000 cells per well and cultured for 3 days or until fully confluent. Thirty thousand HDMECs (P5-10) were then seeded on top of the NHDF monolayer and cultured for 1 day in 0.5 mL complete endothelial cell growth medium V2, after which the medium was changed to basal endothelial cell growth medium V2 supplemented with 1% fetal bovine serum and 10% media conditioned by WT or CST B/B iPS-RPE cells for 24 hours. In some experiments, recombinant cystatin C (Thermo Fisher Scientific) was added to the preconditioned media at a concentration of 50 ng/mL, resulting in a final co-culture concentration of 5 ng/mL. Co-cultures were maintained for 5 days with one media exchange, after which cells were fixed in ice-cold 70% (v/v) ethanol for 30 minutes, blocked with 1% BSA in PBS for 30 minutes and immunostained against CD31 using a mouse anti-CD31 antibody (9498; Abcam) diluted to 1 µg/mL in block buffer for 1 hour. Cells were washed three times in PBS and incubated with a goat anti-mouse IgG-AP antibody (3562; Sigma-Aldrich) diluted 1:500 in block buffer for 1 hour. Cells were finally washed three times in distilled H_2_O, developed using SigmaFast BCIP/NBT alkaline phosphatase substrate (Sigma-Aldrich) and imaged using a Nikon Diaphot inverted light microscope. Five separate fields were recorded per well and analyzed using AngioQuant software.^32^

### Statistics

Data are presented as means ± SD of at least three independent biological replicate experiments. Statistical analyses were performed using SigmaPlot version 13 (Systat Software, Inc). Two-tailed Student's *t*-test was used to compare differences between mean values, with *P* values < 0.05 considered statistically significant.

## Results

### Gene Editing and Differentiation of iPSCs to RPE

**Figure 1.**
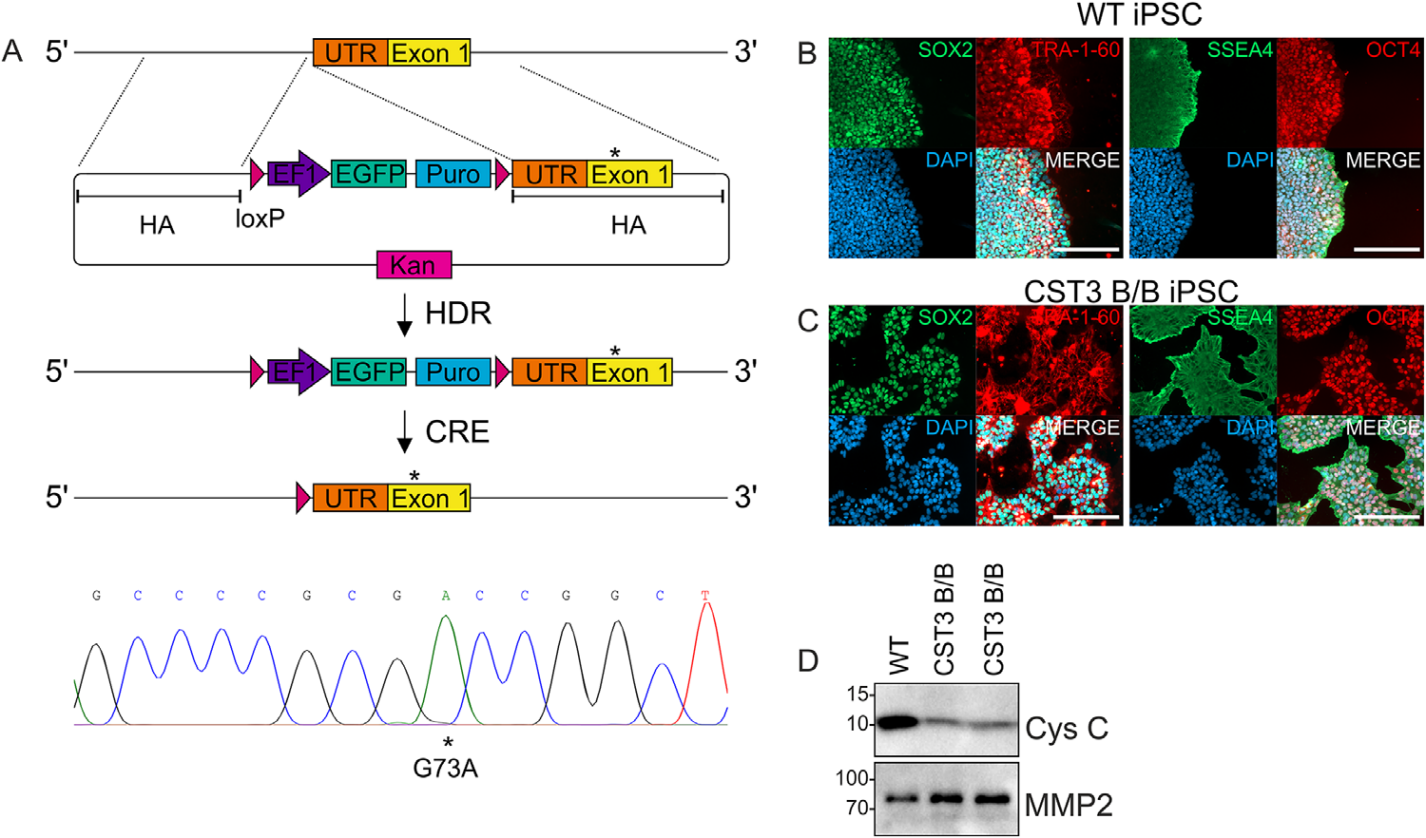
Bi-allelic gene editing of iPSCs to encode variant B cystatin C. **(A)** Overall schematic of strategy used for gene editing of iPSCs (top). Sequencing of the CST3 gene confirmed a homozygous G73A edit (bottom). Following gene editing, pluripotency staining of WT **(B)** and edited **(C)** cells using antibodies against SOX2, TRA-1-60, SSEA4, and OCT4 demonstrated that the editing had not affected the pluripotent state of the cells. *Scale bars* = 200 µm. **(D)** Western blot analysis of conditioned media from two separate clones showed that cystatin C secretion was strongly reduced. Images shown are representative of 3 individual experiments.

Variant B cystatin C expression has previously been studied primarily as overexpression in cell lines such as ARPE19 with reduced protein secretion being one of the notable findings,^23^ which was also evidenced when evaluating endogenous expression in primary fibroblasts.^22^ Because the availability of cells homozygous for variant B cystatin C is low and comes with a high degree of variation between donor cells, we wanted to establish a defined platform for RPE cells homozygous for either WT or variant B cystatin C. The method for using CRISPR/Cas9 gene editing of iPSCs to generate a biallelic edit in the CST3 gene encoding an A25T mutation in the protein coding sequence has been described previously,^26^ with a brief overview of the editing strategy shown in Figure 1A. Gene-edited iPSCs did not display any noticeable difference in morphology compared with WT cells, and to ensure that the editing process did not interfere with the pluripotent state of the cells, immunostaining of selected pluripotency markers SSEA4, OCT4, SOX2, and TRA-1-60 was performed immediately before induction of differentiation (Figs. 1B–C), with both WT and edited iPSCs displaying clear expression of all four markers. Secretion of cystatin C from gene-edited cells was confirmed to be significantly reduced compared with WT controls, whereas secretion of matrix metalloproteinase 2 MMP2, a protein known to be abundantly expressed by iPSCs,^33^ was slightly elevated (Fig. 1D).

**Figure 2.**
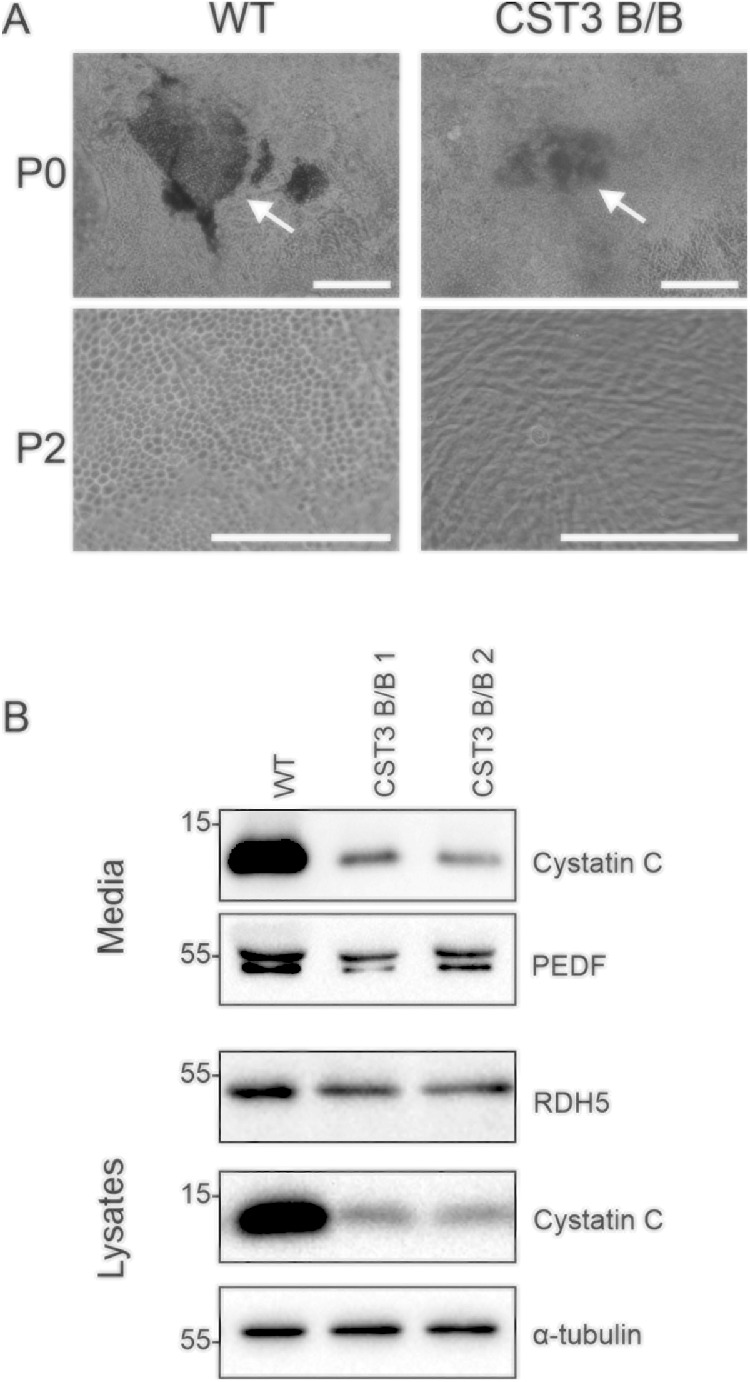
Differentiation of gene-edited iPSCs to RPE cells. **(A)** Hyperpigmented patches of RPE cells after differentiation were excised at *P0* (upper panels) and further expanded. Lower panels show cells at *P2*. *Arrows,* pigmented patches before excision during P0; *Scale bars* = 250 µm. **(B)** Western blot analysis of conditioned media and whole cells lysates confirmed reduced cystatin C expression and secretion, as well as expression of RDH5 and secretion of PEDF. Images shown are representative of three individual experiments.

Directed differentiation of iPSCs into RPE-like cells was achieved by using a previously described protocol,^27^ with pigmented patches starting to appear after approximately 1 month of culture. At this stage, it became apparent that the pigmentation of gene-edited cells was not as strong as seen in WT cells (Fig. 2A). Although WT cells displayed defined black spots of hyperpigmented cells, the spots seen in edited cell cultures were fainter with diffuse borders. Patches were manually excised and subcultured on newly prepared matrigel-coated plates and after approximately 1 month of further culture WT and edited iPS-RPE cells had both established tight monolayers of cells. Only WT cells displayed sections with the cobblestone phenotype characteristic for RPE (Fig. 2A), and pigmentation was noticeably stronger in WT cells, phenotypes which continued to be observed during following passages. Expression of visual cycle protein and RPE marker RDH5 was confirmed by Western blot analysis for both WT and edited cells, as well as secretion of PEDF (Fig. 2B). Two individual gene-edited clones were differentiated separately and analyzed by functional assays throughout this study.

### Effect of Reduced Cystatin C Expression on Monolayer Polarity

**Figure 3.**
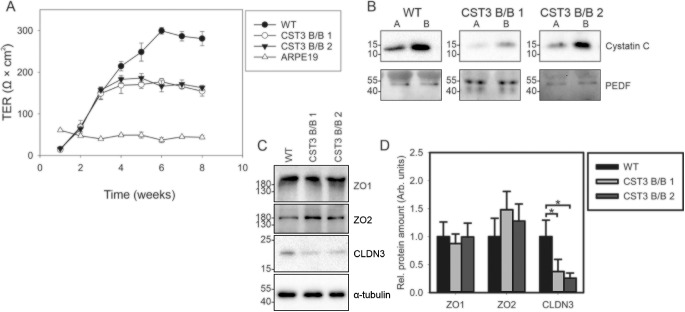
Reduced TER in CST3 B/B iPS-RPE cells. **(A)** TER of WT and gene-edited iPS-RPE cells seeded on transwell inserts was monitored over 8 weeks. **(B)** Conditioned media from the upper and lower wells of transwell inserts, corresponding to apical and basolateral secretion, were analyzed by Western blot for cystatin C and PEDF secretion. *A,* apical secretion; *B,* basolateral secretion. **(C)** Expression of tight junction proteins ZO1, ZO2, and claudin 3 was confirmed by Western blot. Images shown are representative of three individual experiments. **(D)** Densitometric analysis of tight junction protein content from whole-cell lysates shown in panel **C**. n = 3; *, *P* ≤ 0.05.

To evaluate whether editing of cystatin C gene had any effect on polarized secretion and TER, cells were cultured on transwell inserts allowing for separate collection of media conditioned to the apical or basolateral side of the monolayer. The human ARPE19 spontaneously immortalized RPE cell line was cultured in a similar manner and used for comparison. As seen in Figure 3A, ARPE19 TER quickly reached its highest point of approximately 50 Ω × cm^2^ after just 1 week in culture, a value consistent with previous studies,^24^^,^^34^ whereas the iPS-derived RPE cells slowly matured toward significantly higher levels. CST3 B/B cells reached their maximum levels of approximately 150–200 Ω × cm^2^ after 4 weeks in culture, whereas non-edited cells displayed maximum values approaching 300 Ω × cm^2^ after 6 weeks in culture. Although these levels are lower than those observed for *in vitro* cultures of primary RPE cells,^34^^–^^36^ they are a clear improvement over the ARPE19 model cell line, prompting us to investigate whether any polarization of secreted proteins could be observed. Western blot analysis of proteins secreted from the apical and basolateral side of the monolayer showed a clear polarization of cystatin C secretion from both WT and CST3 B/B cells (Fig. 3B). To demonstrate that this predominant basolateral secretion was not a global feature of the cells, we also probed the membranes against PEDF, a protein previously shown to be predominantly secreted apically from RPE cells, where the abundance was not significantly different between the two sides (Fig. 3B). TER is known to be a function of tight junction strands^37^ comprised in RPE of proteins such as ZO1, ZO2, claudins, and occludin, prompting us to investigate whether CST3 B/B cells displayed reduced expression of these proteins. Western blot analysis of iPS-RPE whole cell lysates showed no significant difference in expression of tight junction proteins ZO1 or ZO2; however, claudin-3 expression was significantly reduced in CST3 B/B cells (Figs. 3C–D).

### Effect of Reduced Cystatin C Expression on ECM Composition

**Figure 4.**
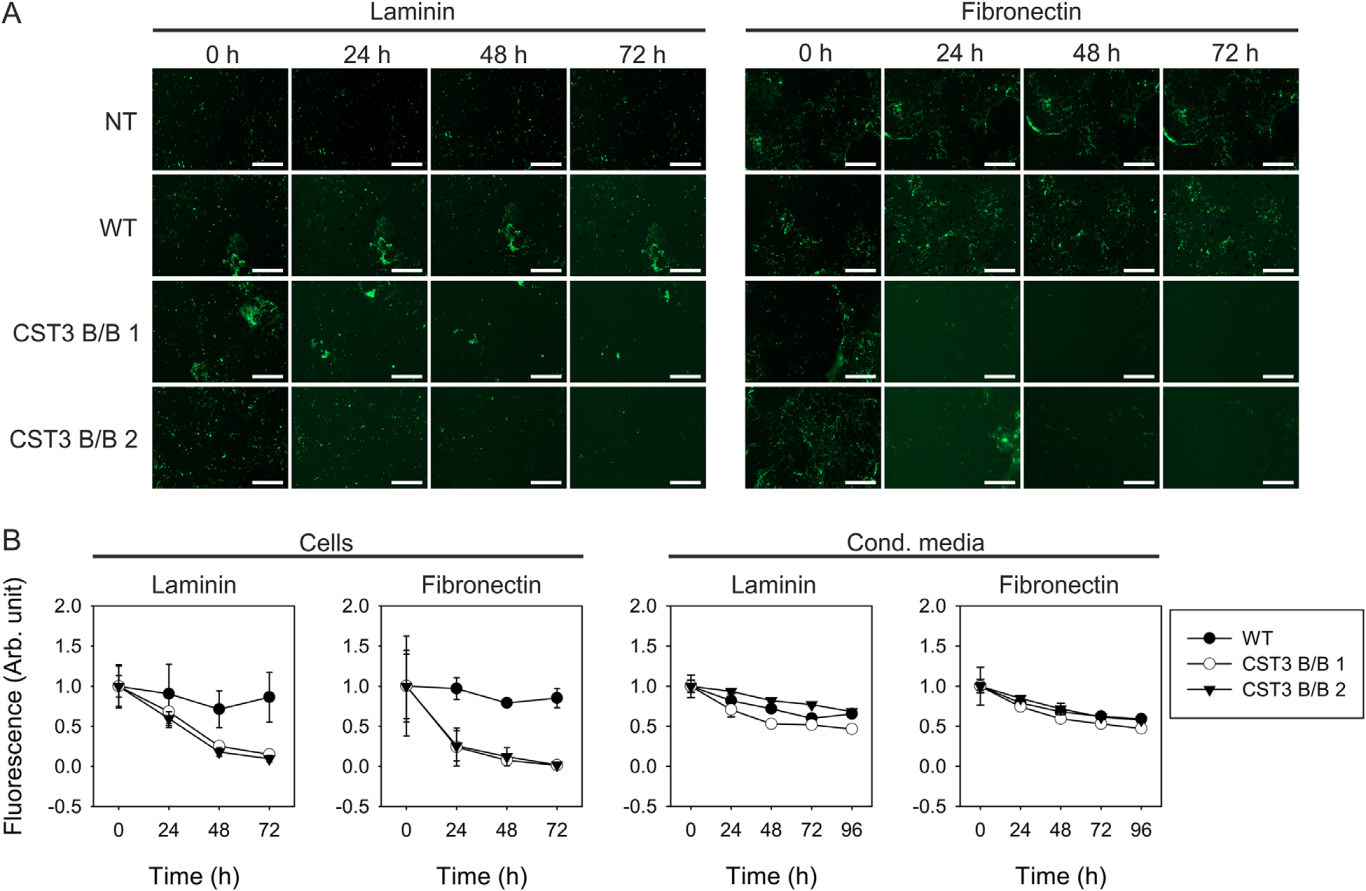
Faster degradation of ECM by CST3 B/B iPS-RPE cells. **(A)** Degradation of fluorescently labeled laminin and fibronectin incorporated into NIH3T3 fibroblast ECM 3D-matrix by iPS-RPE cells or conditioned media was analyzed by fluorescence microscopy. *NT,* no treatment; *Scale bars* = 200 µm. **(B)** Quantitative image analysis of total integrated fluorescence. Gene-edited iPS-RPE cells were able to degrade both laminin and fibronectin faster than WT cells, although no difference in degradation rate could be seen when subjecting the labeled ECM to conditioned media.

Cystatin C is one of the most abundantly expressed proteins in the RPE^13^ and mainly acts as an inhibitor of various proteases, many of which are known to cleave commonly expressed ECM proteins such as laminin and fibronectin.^7^ To evaluate whether the reduced expression levels of cystatin C in our gene-edited iPS-RPE cells were associated with a higher rate of ECM degradation, we first incorporated fluorescently labeled laminin and fibronectin into ECM deposited onto matrigel-coated plates by mouse fibroblast cells. Cells were then lysed via NH_4_OH exposure, leaving the deposited ECM intact. The iPS-RPE cells were seeded on top and cultured for up to 3 days with daily monitoring by fluorescence microscopy. As seen in Figures 4A-B, gene-edited iPS-RPE cells were able to degrade both laminin and fibronectin in the deposited ECM significantly faster than the WT cells. Because cystatin C is a potent inhibitor of secreted proteases, we suspected that this effect might have been due to the loss of cystatin C regulatory effect in the RPE secretome. However, incubation of fluorescent ECM matrix with media conditioned to either WT or gene-edited iPS-RPE cells for up to 3 days yielded no discernible difference in rate of degradation (Fig. 4B). From these results, it became clear that the increased rate of laminin and fibronectin degradation required the physical contact to the RPE cells.

### Effect on Cell Adherence and Migration on ECM

**Figure 5.**
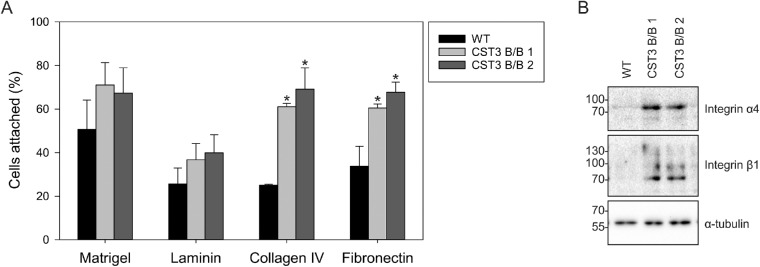
Increased adhesion to ECM-coated wells by CST3 B/B iPS-RPE cells. **(A)** The iPS-RPE cells were subjected to cell adhesion assays on wells coated with matrigel, laminin, collagen IV, or fibronectin. A significantly larger proportion of gene-edited cells were able to attach to wells coated with collagen IV and fibronectin, compared with WT cells, after 1 hour. No significant difference could be seen when seeding on wells coated with matrigel or laminin. n = 3; *, P ≤ 0.05. **(B)** Western blot analysis of iPS-RPE whole cell lysates shows increased expression of integrin α4 and β1 in CST3 B/B cells. Images shown are representative of three individual experiments.

To investigate whether cellular adhesion and migration are affected in gene-edited cells expressing variant B cystatin C, tissue culture plates were coated with common ECM proteins laminin, collagen IV and fibronectin, as well as matrigel, which is comprised by a complex mixture of proteins. The iPS-RPE cells loaded with the fluorescent dye calcein AM were seeded onto these plates, followed by washing after 1 hour of allowing the cells to attach to the well surface. Gene-edited cells adhered to the plates more efficiently than WT cells when seeded on collagen IV and fibronectin (Fig. 5A). The difference in rate of adherence to matrigel and laminin were not statistically significant between WT and edited iPS-RPE. Cellular adherence to ECM is largely dependent on interactions with integrin receptors, and we detected a notable increase in expression of integrin α4 and β1 in edited iPS-RPE cells (Fig. 5B).

**Figure 6.**
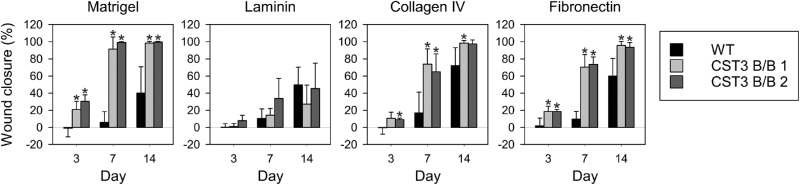
Faster wound closure of CST3 B/B iPS-RPE cells. Wound healing assays were conducted with iPS-RPE cells cultured on matrigel, laminin, collagen IV, or fibronectin. Wound closure of gene-edited iPS-RPE cells was significantly higher after 7 days when cultured on matrigel, collagen IV and fibronectin. No significant difference could be seen between WT and gene edited iPS-RPE cells when cultured on laminin. n = 3; *, *P* ≤ 0.05.

The ability of cells to migrate on the different surfaces was also tested by allowing the cells to establish confluent monolayers on the different coatings and inducing a wound using a sterile pipette tip. Frequent monitoring by light microscopy revealed that gene-edited cells migrated significantly faster on all tested surfaces except laminin-coated plates (Fig. 6). The difference was especially striking when cultured on matrigel for 7 days, at which edited cells had achieved near complete closure while WT cells had barely begun to close the wound.

### Reduced Cystatin C in Gene Edited iPS-RPE-Conditioned Media Supports a Pro-Angiogenic Environment

**Figure 7.**
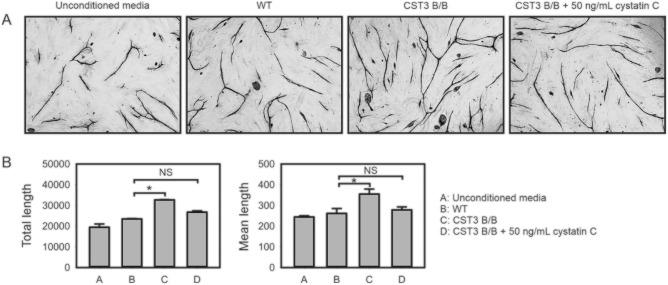
Increased angiogenesis supported by media conditioned by CST3 B/B iPS-RPE cells**. (A)** NHDF/HDMEC co-cultures were stimulated with media conditioned by WT or CST3 B/B iPS-RPE cells, followed by microvascular staining with anti-CD31 antibody and analysis by light microscopy. Images shown are representative of fields from three separate experiments. **(B)** Quantitative analysis of microvascular networks from NHDF/HDMEC co-culture experiments. n = 3; *, *P* ≤ 0.05.

Media conditioned by WT or gene edited iPS-RPE cells collected after 24 hours was supplemented to the culture media used in a NHDF/HDMEC co-culture angiogenesis assay to investigate whether the changes in secretion seen in the edited cells were better at promoting microvascular tube formation. Indeed, we found that co-cultures using the media conditioned by cystatin C gene edited iPS-RPE cells achieved a clear difference in HDMEC tubular morphology, with significantly longer tubules formed (Fig. 7). This effect could be offset by adding exogenous cystatin C to the media at a concentration of 50 ng/mL, which is approximately the concentration we previously detected in confluent cultures of primary fetal RPE cells after 24 hours.^36^

## Discussion

The gene-edited iPS-RPE cell lines presented reduced cystatin C secretion, consistent with previous studies on variant B cystatin C genotype in cultured cells,^22^^,^^23^^,^^38^ serum plasma,^39^ or cerebrospinal fluid,^40^^,^^41^ with one exception—a study by Nguyen et al.^42^ that did not observe a functional effect on secretion rate conferred by the mutation when overexpressed in HEK293T and non-differentiated ARPE19 cells. This study also noted significantly lower levels of intracellular cystatin C in gene-edited cells, highlighting that expression level was affected. An increased risk for developing AMD, as well as other degenerative diseases such as Alzheimer's disease^15^^,^^17^^–^^20^ has been attributed to this mutation, making functional studies on how the cells behave in defined conditions valuable. At the same time, the Beaver Dam Eye Study identified a correlation between increased cystatin C serum levels and AMD pathology,^43^ further complicating the functional relationship between cystatin C and AMD. Elevated cystatin C serum concentration was also described as a risk factor for other disease conditions, such as cardiovascular disease^44^^,^^45^ and progression to prediabetes.^46^ However, significantly reduced serum cystatin C levels, which had been correlated with variant haplotypes,^47^^,^^48^ were highlighted in association with atherosclerotic lesions, dilated aorta and focal progression of coronary artery disease.^47^^,^^49^^,^^50^ These data on involvement of cystatin C proteostasis in cardiovascular disease suggested that cystatin C deficiency alters arterial structures through impaired regulation of proteolysis of the arterial ECM.

Both WT and edited iPSCs could be differentiated to RPE phenotype using a previously described targeted differentiation protocol.^27^ We favored a targeted instead of spontaneous differentiation protocol mainly due to being able to maintain consistency between experiments. Hyperpigmented patches of RPE-like cells appeared at identical time points for both WT and edited cells, which could be manually excised and further subcultured for multiple passages. Clear differences in morphology were observed between WT and CST3 B/B iPS-RPE cells, although immunoblot analysis of whole-cell lysates showed no significant difference in expression of visual cycle and tight junction proteins. Gene-edited cells appeared smaller and less pigmented after differentiation, and adopted less regular hexagonal patterns, which could point to RPE developmental problems.

The overall process for obtaining genetically defined RPE cells described here could easily be applied towards functional studies of other genotypes associated with retinal diseases, and as the gene editing is done at the iPSC stage, the cells could also hypothetically be differentiated to other relevant cell types.

The level of reduction in cystatin C secretion seen in our gene-edited cells was dramatic, but consistent between two separate cell lines generated. A previous study in primary CST3 A/A, A/B and B/B skin fibroblast cells demonstrated high level of variation in cystatin C secretion between donors of the same genotype,^22^ highlighting the value of using nonedited cells, which are otherwise genetically the same, as controls. However, in their study the expression levels seen in whole cell lysates were not significantly affected by the B/B genotype, whereas we observed a clear reduction in expression levels. It is possible that this effect is cell-type dependent, or that the remaining scar sequence from gene editing located upstream of exon 1 (Fig. 1A) affects the level of protein expression, as a previous study has identified this region to be sensitive to modifications,^51^ thus accentuating the reduction of secretion.

Because cystatin C is a main regulator for multiple cysteine proteases known to degrade ECM proteins, the effects of reduced protein expression and secretion have on cellular interaction with ECM structures was our main focus in this study. Cystatin C is predominantly secreted from the basolateral side of the RPE monolayer, which is located directly adjacent to the collagen-rich BrM and is therefore likely to in part regulate BrM maintenance and remodeling. With both age and AMD progression, the BrM is known to experience thickening,^52^ a likely result of progressive loss of proteostatic control. Whether cystatin C plays a role in this process is yet to be established.

Changes in migration and adhesion of gene-edited iPS-RPE cells appear to be substrate dependent, as neither was significantly altered compared with WT cells when cultured on laminin-coated wells. This suggests active mechanistic functions being affected, rather than general cellular properties. As laminin is the predominant component of matrigel (approximately 60% [w/w]), we were surprised to see that the migration rate on matrigel was dramatically increased in edited iPS-RPE cells, whereas no significant difference in adhesion compared with WT cells was seen. Experiments conducted on collagen IV, which is the second-most abundant component of matrigel (approximately 30% [w/w]), resulted in clear alteration of both adhesion and migration in edited cells.

Analyzing whole-cell lysates, we detected a clear increase in expression of multiple integrins. These transmembrane receptor proteins form heterodimers in different combinations that provide interactions between the intracellular cytoskeleton and extracellular ECM molecules, thereby playing key roles in cellular adhesion.^53^ Specifically, we detected marked increases in integrins α4 and β1 expression. The integrin α4β1 heterodimer recognizes fibronectin,^54^ to which edited iPS-RPE cells displayed increased adhesion, although both chains are also involved in other combinations that mediate interaction with laminins and collagens. It is possible that integrin expression becomes upregulated in response to increased degradation rate of ECM coatings on the tissue culture wells, which would explain the increased rate of adhesion to fresh coatings when subcultured.

Average content of tight junction protein claudin-3 was significantly reduced in our gene edited iPS-RPE cells. This is the second most abundantly expressed claudin in RPE cells,^55^ and crucially, depletion of claudins has been shown to have the potential to drastically disrupt TER in cultured RPE cells.^55^ This may in part explain why our edited iPS-RPE cells also display a significant reduction of TER after 6 weeks in culture.

To functionally test the specific link between our model and exudative AMD phenotype, we investigated how changes in cystatin C expression and secretion affect microvascular tube formation in an NHDF/HDMEC co-culture angiogenesis model. The dysregulated secretion seen in our gene-edited cells resulted in an increased ability for HDMECs to form vessels when cultured in media preconditioned by the variant B cystatin C iPS-RPE cells. As the effect was eliminated when supplementing the media with exogenous cystatin C, this demonstrates a functional association between cystatin C and angiogenesis, further underlining the clinical relevance of cystatin C as an effector molecule in the context of wet AMD.

In conclusion, reduced expression of cystatin C in iPS-derived RPE cells had clear effects on cells’ behavior and interaction with ECM. In this study, reduced expression was induced via CRISPR/Cas9 gene editing, but reduced protein levels have previously been described in human fetal RPE cells cultured on ECM subjected to advanced glycation end products and a general decline with age in posterior RPE segments.^36^ The evidence presented provides a functional link between reduced cystatin C expression and RPE invasiveness in a pro-angiogenic environment relevant for wet AMD pathology, affording mechanistic data explaining the functional relationship between cystatin C and cellular ECM interactions.

## References

[1] Strauss O. The retinal pigment epithelium in visual function. *Physiol Rev.* 2005; 85: 845–881. 15987797 10.1152/physrev.00021.2004

[2] Ambati J, Fowler BJ. Mechanisms of age-related macular degeneration. *Neuron.* 2012; 75: 26–39. 22794258 10.1016/j.neuron.2012.06.018 PMC3404137

[3] Bhutto I, Lutty G Understanding age-related macular degeneration (AMD): relationships between the photoreceptor/retinal pigment epithelium/Bruch's membrane/choriocapillaris complex. *Mol Aspects Med.* 2012; 33: 295–317. 22542780 10.1016/j.mam.2012.04.005 PMC3392421

[4] Kay P, Yang YC, Paraoan L Directional protein secretion by the retinal pigment epithelium: roles in retinal health and the development of age-related macular degeneration. *J Cell Mol Med.* 2013; 17: 833–843. 23663427 10.1111/jcmm.12070 PMC3822888

[5] Turk V, Stoka V, Vasiljeva O et al. Cysteine cathepsins: from structure, function and regulation to new frontiers. *Biochim Biophys Acta.* 2012; 1824: 68–88. 22024571 10.1016/j.bbapap.2011.10.002 PMC7105208

[6] Rossi A, Deveraux Q, Turk B, Sali A Comprehensive search for cysteine cathepsins in the human genome. *Biol Chem.* 2004; 385: 363–372. 15195995 10.1515/BC.2004.040

[7] Fonovic M, Turk B Cysteine cathepsins and extracellular matrix degradation. *Biochim Biophys Acta.* 2014; 1840: 2560–2570. 24680817 10.1016/j.bbagen.2014.03.017

[8] Vizovisek M, Fonovic M, Turk B Cysteine cathepsins in extracellular matrix remodeling: Extracellular matrix degradation and beyond. *Matrix Biol.* 2019; 75-76: 141–159. 29409929 10.1016/j.matbio.2018.01.024

[9] Kramer L, Turk D, Turk B The future of cysteine cathepsins in disease management. *Trends Pharmacol Sci.* 2017; 38: 873–898. 28668224 10.1016/j.tips.2017.06.003

[10] Reiser J, Adair B, Reinheckel T Specialized roles for cysteine cathepsins in health and disease. *J Clin Invest.* 2010; 120: 3421–3431. 20921628 10.1172/JCI42918 PMC2947230

[11] Stoka V, Turk V, Turk B Lysosomal cathepsins and their regulation in aging and neurodegeneration. *Ageing Res Rev.* 2016; 32: 22–37. 27125852 10.1016/j.arr.2016.04.010

[12] Ida H, Boylan SA, Weigel AL et al. EST analysis of mouse retina and RPE/choroid cDNA libraries. *Mol Vis.* 2004; 10: 439–444. 15257269

[13] Paraoan L, Grierson I, Maden BE Analysis of expressed sequence tags of retinal pigment epithelium: cystatin C is an abundant transcript. *Int J Biochem Cell Biol.* 2000; 32: 417–426. 10762067 10.1016/s1357-2725(99)00143-0

[14] Wistow G, Bernstein SL, Wyatt MK et al. Expressed sequence tag analysis of human RPE/choroid for the NEIBank Project: over 6000 non-redundant transcripts, novel genes and splice variants. *Mol Vis.* 2002; 8: 205–220. 12107410

[15] Butler JM, Sharif U, Ali M et al. A missense variant in CST3 exerts a recessive effect on susceptibility to age-related macular degeneration resembling its association with Alzheimer's disease. *Hum Genet.* 2015; 134: 705–715. 25893795 10.1007/s00439-015-1552-7 PMC4460273

[16] Zurdel J, Finckh U, Menzer G, Nitsch RM, Richard G CST3 genotype associated with exudative age related macular degeneration. *Br J Ophthalmol.* 2002; 86: 214–219. 11815350 10.1136/bjo.86.2.214 PMC1771004

[17] Bertram L, McQueen MB, Mullin K, Blacker D, Tanzi RE Systematic meta-analyses of Alzheimer disease genetic association studies: the AlzGene database. *Nat Genet.* 2007; 39: 17–23. 17192785 10.1038/ng1934

[18] Crawford FC, Freeman MJ, Schinka JA et al. A polymorphism in the cystatin C gene is a novel risk factor for late-onset Alzheimer's disease. *Neurology.* 2000; 55: 763–768. 10993992 10.1212/wnl.55.6.763

[19] Finckh U, von der Kammer H, Velden J et al. Genetic association of a cystatin C gene polymorphism with late-onset Alzheimer disease. *Arch Neurol.* 2000; 57: 1579–1583. 11074789 10.1001/archneur.57.11.1579

[20] Hua Y, Zhao H, Lu X, Kong Y, Jin H Meta-analysis of the cystatin C(CST3) gene G73A polymorphism and susceptibility to Alzheimer's disease. *Int J Neurosci.* 2012; 122: 431–438. 22435454 10.3109/00207454.2012.672502

[21] Ohno-Matsui K. Parallel findings in age-related macular degeneration and Alzheimer's disease. *Prog Retin Eye Res.* 2011; 30: 217–238. 21440663 10.1016/j.preteyeres.2011.02.004

[22] Benussi L, Ghidoni R, Steinhoff T et al. Alzheimer disease-associated cystatin C variant undergoes impaired secretion. *Neurobiology of Disease.* 2003; 13: 15–21. 12758063 10.1016/s0969-9961(03)00012-3

[23] Paraoan L, Ratnayaka A, Spiller DG, Hiscott P, White MR, Grierson I Unexpected intracellular localization of the AMD-associated cystatin C variant. *Traffic.* 2004; 5: 884–895. 15479453 10.1111/j.1600-0854.2004.00230.x

[24] Dunn KC, Aotaki-Keen AE, Putkey FR, Hjelmeland LM ARPE-19, a human retinal pigment epithelial cell line with differentiated properties. *Exp Eye Res.* 1996; 62: 155–169. 8698076 10.1006/exer.1996.0020

[25] Leach LL, Clegg DO. Concise review: making stem cells retinal: methods for deriving retinal pigment epithelium and implications for patients with ocular disease. *Stem Cells.* 2015; 33: 2363–2373. 25809736 10.1002/stem.2010

[26] Supharattanasitthi W, Carlsson E, Sharif U, Paraoan L CRISPR/Cas9-mediated one step bi-allelic change of genomic DNA in iPSCs and human RPE cells in vitro with dual antibiotic selection. *Sci Rep.* 2019; 9: 174. 30655567 10.1038/s41598-018-36740-2 PMC6336765

[27] Buchholz DE, Pennington BO, Croze RH, Hinman CR, Coffey PJ, Clegg DO Rapid and efficient directed differentiation of human pluripotent stem cells into retinal pigmented epithelium. *Stem Cells Transl Med.* 2013; 2: 384–393. 23599499 10.5966/sctm.2012-0163 PMC3667566

[28] Schneider CA, Rasband WS, Eliceiri KW NIH Image to ImageJ: 25 years of image analysis. *Nat Methods.* 2012; 9: 671–675. 22930834 10.1038/nmeth.2089 PMC5554542

[29] Chlenski A, Guerrero LJ, Salwen HR et al. Secreted protein acidic and rich in cysteine is a matrix scavenger chaperone. *PLoS One.* 2011; 6: e23880. 21949685 10.1371/journal.pone.0023880 PMC3174944

[30] Postovit LM, Seftor EA, Seftor RE, Hendrix MJ A three-dimensional model to study the epigenetic effects induced by the microenvironment of human embryonic stem cells. *Stem Cells.* 2006; 24: 501–505. 16293574 10.1634/stemcells.2005-0459

[31] Hetheridge C, Mavria G, Mellor H Uses of the in vitro endothelial-fibroblast organotypic co-culture assay in angiogenesis research. *Biochem Soc Trans.* 2011; 39: 1597–1600. 22103493 10.1042/BST20110738

[32] Niemisto A, Dunmire V, Yli-Harja O, Zhang W, Shmulevich I Robust quantification of in vitro angiogenesis through image analysis. *IEEE Trans Med Imaging.* 2005; 24: 549–553. 15822812 10.1109/tmi.2004.837339

[33] Bock C, Kiskinis E, Verstappen G et al. Reference Maps of human ES and iPS cell variation enable high-throughput characterization of pluripotent cell lines. *Cell.* 2011; 144: 439–452. 21295703 10.1016/j.cell.2010.12.032 PMC3063454

[34] Ablonczy Z, Dahrouj M, Tang PH et al. Human retinal pigment epithelium cells as functional models for the RPE in vivo. *Invest Ophthalmol Vis Sci.* 2011; 52: 8614–8620. 21960553 10.1167/iovs.11-8021 PMC3208409

[35] Benedicto I, Lehmann GL, Ginsberg M et al. Concerted regulation of retinal pigment epithelium basement membrane and barrier function by angiocrine factors. *Nat Commun.* 2017; 8: 15374. 28524846 10.1038/ncomms15374 PMC5454459

[36] Kay P, Yang YC, Hiscott P, Gray D, Maminishkis A, Paraoan L Age-related changes of cystatin C expression and polarized secretion by retinal pigment epithelium: potential age-related macular degeneration links. *Invest Ophthalmol Vis Sci.* 2014; 55: 926–934. 24458156 10.1167/iovs.13-13239 PMC11980428

[37] Weber CR. Dynamic properties of the tight junction barrier. *Ann N Y Acad Sci.* 2012; 1257: 77–84. 22671592 10.1111/j.1749-6632.2012.06528.x PMC3687038

[38] Ratnayaka A, Paraoan L, Spiller DG et al. A dual Golgi- and mitochondria-localised Ala25Ser precursor cystatin C: an additional tool for characterising intracellular mis-localisation leading to increased AMD susceptibility. *Exp Eye Res.* 2007; 84: 1135–1139. 16635487 10.1016/j.exer.2006.01.030

[39] Noto D, Cefalu AB, Barbagallo CM et al. Cystatin C levels are decreased in acute myocardial infarction: effect of cystatin C G73A gene polymorphism on plasma levels. *Int J Cardiol.* 2005; 101: 213–217. 15882666 10.1016/j.ijcard.2004.03.018

[40] Maetzler W, Schmid B, Synofzik M et al. The CST3 BB genotype and low cystatin C cerebrospinal fluid levels are associated with dementia in Lewy body disease. *J Alzheimers Dis.* 2010; 19: 937–942. 20157249 10.3233/JAD-2010-1289

[41] Yamamoto-Watanabe Y, Watanabe M, Jackson M et al. Quantification of cystatin C in cerebrospinal fluid from various neurological disorders and correlation with G73A polymorphism in CST3. *Brain Res.* 2010; 1361: 140–145. 20849835 10.1016/j.brainres.2010.09.033

[42] Nguyen A, Hulleman JD. Evidence of Alternative Cystatin C Signal Sequence Cleavage Which Is Influenced by the A25T Polymorphism. *PLoS One.* 2016; 11: e0147684. 26845025 10.1371/journal.pone.0147684 PMC4741414

[43] Klein R, Knudtson MD, Lee KE, Klein BE Serum cystatin C level, kidney disease markers, and incidence of age-related macular degeneration: the Beaver Dam Eye Study. *Arch Ophthalmol.* 2009; 127: 193–199. 19204238 10.1001/archophthalmol.2008.551 PMC2737458

[44] Melander O, Newton-Cheh C, Almgren P et al. Novel and conventional biomarkers for prediction of incident cardiovascular events in the community. *JAMA.* 2009; 302: 49–57. 19567439 10.1001/jama.2009.943 PMC3090639

[45] Sarnak MJ, Katz R, Stehman-Breen CO et al. Cystatin C concentration as a risk factor for heart failure in older adults. *Ann Intern Med.* 2005; 142: 497–505. 15809461 10.7326/0003-4819-142-7-200504050-00008

[46] Donahue RP, Stranges S, Rejman K, Rafalson LB, Dmochowski J, Trevisan M Elevated cystatin C concentration and progression to pre-diabetes: the Western New York study. *Diabetes Care.* 2007; 30: 1724–1729. 10.2337/dc07-0040 17456840

[47] Eriksson P, Deguchi H, Samnegard A et al. Human evidence that the cystatin C gene is implicated in focal progression of coronary artery disease. *Arterioscler Thromb Vasc Biol.* 2004; 24: 551–557. 14726415 10.1161/01.ATV.0000117180.57731.36

[48] Loew M, Hoffmann MM, Koenig W, Brenner H, Rothenbacher D Genotype and plasma concentration of cystatin C in patients with coronary heart disease and risk for secondary cardiovascular events. *Arterioscler Thromb Vasc Biol.* 2005; 25: 1470–1474. 15860739 10.1161/01.ATV.0000168416.74206.62

[49] Shi GP, Sukhova GK, Grubb A et al. Cystatin C deficiency in human atherosclerosis and aortic aneurysms. *J Clin Invest.* 1999; 104: 1191–1197. 10545518 10.1172/JCI7709 PMC409823

[50] Sukhova GK, Wang B, Libby P et al. Cystatin C deficiency increases elastic lamina degradation and aortic dilatation in apolipoprotein E-null mice. *Circ Res.* 2005; 96: 368–375. 15653570 10.1161/01.RES.0000155964.34150.F7

[51] Olafsson I. The human cystatin C gene promoter: functional analysis and identification of heterogeneous mRNA. *Scand J Clin Lab Invest.* 1995; 55: 597–607. 8633184 10.3109/00365519509110259

[52] Booij JC, Baas DC, Beisekeeva J, Gorgels TG, Bergen AA The dynamic nature of Bruch's membrane. *Prog Retin Eye Res.* 2010; 29: 1–18. 19747980 10.1016/j.preteyeres.2009.08.003

[53] Barczyk M, Carracedo S, Gullberg D Integrins. *Cell Tissue Res.* 2010; 339: 269–280. 19693543 10.1007/s00441-009-0834-6 PMC2784866

[54] Chan BM, Elices MJ, Murphy E, Hemler ME Adhesion to vascular cell adhesion molecule 1 and fibronectin. Comparison of alpha 4 beta 1 (VLA-4) and alpha 4 beta 7 on the human B cell line JY. *J Biol Chem.* 1992; 267: 8366–8370. 1373725

[55] Peng S, Rao VS, Adelman RA, Rizzolo LJ Claudin-19 and the barrier properties of the human retinal pigment epithelium. *Invest Ophthalmol Vis Sci.* 2011; 52: 1392–1403. 21071746 10.1167/iovs.10-5984 PMC3101667

